# Relationship between ostracism and psychological crisis vulnerability among Chinese college students: the mediating roles of self-uncertainty and subjective social status

**DOI:** 10.3389/fpsyg.2026.1707544

**Published:** 2026-02-24

**Authors:** Junliang Li, Chunyu Li

**Affiliations:** 1 Key Research Base of Humanities and Social Sciences of the Ministry of Education, Academy of Psychology and Behavior, Tianjin Normal University, Tianjin, China; 2 Faculty of Psychology, Tianjin Normal University, Tianjin, China; 3 College of Teacher Education, Qujing Normal University, Qujing, China

**Keywords:** ostracism, social exclusion, psychological crisis vulnerability, self-uncertainty, subjective social status, Chinese college students

## Abstract

**Introduction:**

Ostracism is a prevalent interpersonal stressor among college students and has been consistently associated with adverse mental health outcomes. However, limited research has examined the psychological mechanisms through which ostracism increases vulnerability to psychological crisis. Drawing on the need–threat model, uncertainty–identity theory, and social comparison perspectives, the present study aimed to establish a formal multiple mediation model to clarify how ostracism contributes to psychological crisis vulnerability among Chinese college students.

**Methods:**

A total of 758 Chinese college students were recruited from four comprehensive universities located in economically diverse regions of China, including developed areas in Eastern China and less developed areas in Western China. Participants represented a wide range of majors, including STEM, humanities and social sciences, and arts-related programs. Self-report measures assessed ostracism, self-uncertainty, subjective social status, and psychological crisis vulnerability. Mediation analyses were conducted to test the independent and sequential mediating roles of self-uncertainty and subjective social status, with gender included as a covariate.

**Results:**

The results showed that ostracism was positively associated with psychological crisis vulnerability. Both self-uncertainty and subjective social status independently mediated this association. In addition, a significant chain mediation effect was identified, such that ostracism was associated with higher self-uncertainty, which in turn predicted lower subjective social status and, consequently, greater psychological crisis vulnerability.

**Discussion:**

By integrating interpersonal, cognitive, and social-status perspectives into a single multiple mediation framework, this study extends existing research on ostracism and mental health. The findings highlight self-uncertainty and subjective social status as key psychological mechanisms linking ostracism to psychological crisis vulnerability and suggest potential targets for prevention and intervention efforts aimed at reducing psychological crises among college students.

## 1 Introduction

Psychological crisis refers to a mental state in which individuals are unable to effectively cope with sudden events or major setbacks due to insufficient internal resources and coping strategies (Caplan and Grunebaum, 1967; Roennfeldt et al., 2024). Psychological crisis vulnerability reflects the extent to which people lack the capacity to withstand such crises (Guo et al., 2018; Parry, 1990). Higher levels of vulnerability are associated with weaker coping and recovery abilities and increase the risk of anxiety, depression, and even suicidal ideation (Zhou et al., 2010). Compared with transient emotional distress, psychological crisis vulnerability reflects a more generalized susceptibility to entering a crisis state across stress contexts, functioning as an upstream risk indicator for early identification and prevention.

In recent years, psychological crises and mental health problems among Chinese college students have become increasingly prominent. Research findings consistently indicate that a substantial proportion of university students experience significant psychological distress, with depression, anxiety, sleep problems, and self-harm–related symptoms being particularly common (Gao et al., 2020). Evidence from large-scale investigations further suggests that these mental health problems constitute a considerable population-level burden (Lin et al., 2025) and have shown persistent or increasing trends over the past decade (Chen et al., 2022). Importantly, existing studies indicate that students' psychological difficulties are not randomly distributed but are closely associated with a range of psychosocial stressors, including academic pressure, sleep quality problems, low self-esteem, and internet-related addictive behaviors (Han et al., 2025). These findings underscore the urgency of identifying modifiable risk factors that may contribute to heightened psychological crisis vulnerability in this population. This concern is particularly salient during emerging adulthood, when identity development is ongoing and peer relationships serve as a central source of belonging, evaluation, and self-definition (LoParo et al., 2023). In collectivistic cultural contexts such as China, where peer acceptance and social harmony are highly valued, interpersonal exclusion may be particularly salient for self-related evaluations and perceived social standing.

Despite the growing literature on interpersonal adversity and mental health, several gaps remain in prior research. Existing studies have primarily documented associations between ostracism and negative outcomes such as depression, anxiety, aggression, and suicidal ideation (Baumeister et al., 2005; Chen et al., 2020; Niu et al., 2023; Yang et al., 2021), while relatively less attention has been paid to psychological crisis vulnerability as an integrative risk outcome. Moreover, although theoretical perspectives (e.g., the need–threat model) highlight ostracism as a potent interpersonal stressor that threatens fundamental needs (Williams, 2009), empirical work has rarely clarified how ostracism translates into heightened crisis vulnerability through specific psychological processes. Prior research has separately linked self-uncertainty (Van den Bos, 2009; Hogg, 2007, 2009) and subjective social status (Adler et al., 2000; Kraus et al., 2012) to mental health and stress-related outcomes (Cundiff and Matthews, 2017; Zell et al., 2018), yet these mechanisms are typically examined in isolation rather than within a unified framework explaining crisis vulnerability.

These gaps underscore the need to identify modifiable interpersonal and psychological risk processes that may heighten psychological crisis vulnerability in higher education. Among these processes, social experiences of inclusion vs. exclusion are particularly important during emerging adulthood, when peer relationships play a central role. To address these gaps, the present study focuses on ostracism as a key interpersonal stressor and examines how it contributes to psychological crisis vulnerability among Chinese college students through two interconnected psychological mechanisms: self-uncertainty and subjective social status.

## 2 Literature review

### 2.1 Ostracism and psychological crisis vulnerability

Ostracism refers to being ignored, rejected, or excluded by others or groups, which undermines individuals' sense of belonging and social connection (MacDonald and Leary, 2005; Fu, 2021). According to the need-threat model, ostracism threatens four fundamental needs in social contexts—belongingness, self-esteem, control, and meaningful existence (Williams, 2009). When these needs are chronically thwarted, individuals are more likely to experience psychological distress and crisis.

Building on this model, the interpersonal theory of suicide proposes that thwarted belongingness and perceived burdensomeness are core precursors of suicidal ideation (Chu et al., 2017). Empirical research has shown that peer rejection and victimization are strongly associated with self-harm and suicidal behaviors in adolescence (Brunner et al., 2014) and that feeling socially excluded can trigger suicidal thoughts because life appears meaningless without acceptance (Chen et al., 2020). These findings suggest that ostracism is not only a painful social experience, but also a potent risk factor for psychological crisis vulnerability among college students.

However, previous work has mainly demonstrated the existence of associations between ostracism and negative mental health outcomes such as depression, anxiety, aggression, and suicidal ideation (Baumeister et al., 2005; Poon and Chen, 2014; Yang et al., 2021), while paying relatively less attention to the underlying psychological processes that explain why some ostracized students become especially vulnerable to crisis. Two such processes that may be particularly important are self-uncertainty and subjective social status. Together, these findings suggest that ostracism may function as a distal interpersonal stressor that initiates a cascade of psychological processes, ultimately heightening vulnerability to psychological crisis.

### 2.2 Mediating role of self-uncertainty

Self-uncertainty refers to a subjective sense of doubt or instability regarding one's self-concept, core values, and life direction (Van den Bos, 2009). Uncertainty–identity theory argues that when people feel uncertain about themselves, they experience discomfort and a strong motivation to reduce uncertainty, often through social identification (Hogg, 2007, 2009).

Ostracism can pose a serious threat to self-certainty. When college students are ignored or rejected by peers, they feel lonely, devalued, and not accepted, which increases the likelihood of suicidal ideation (Chen et al., 2020). Such adverse social experiences may erode a coherent sense of self and intensify doubts about one's worth and future (Heine et al., 2006; Jiang et al., 2020; Wagoner and Hogg, 2024). Individuals with high self-uncertainty are more prone to anxiety and depression when facing stressors, partly because they lack a stable self-structure to guide coping (Boelen et al., 2014; Luhmann et al., 2011). Recent research further suggests that self-uncertainty undermines psychological resilience and may increase vulnerability to psychological crises (Allan et al., 2023).

Despite these theoretical and empirical indications, few studies have directly examined whether self-uncertainty mediates the association between ostracism and psychological crisis vulnerability. Establishing such a mediational pathway would clarify how interpersonal exclusion is translated into an internal sense of fragility in the face of crises. Thus, self-uncertainty may serve as a key internal mechanism through which experiences of social exclusion are translated into heightened psychological fragility under stress.

### 2.3 Mediating role of subjective social status

Subjective social status refers to individuals' perceived rank in the social hierarchy, formed by comparing themselves with others (Adler et al., 2000; Kraus et al., 2012). Because subjective status inherently involves social comparison, perceiving oneself as being at the bottom of the social ladder is associated with greater stress, fewer resources, and more challenges (Madigan and Daly, 2023). Extensive research has shown that low subjective social status is linked to a wide range of physical and mental health problems, including obesity, cardiovascular disease, sleep disturbances, substance use, depression, and suicidal ideation (Cundiff and Matthews, 2017; Zell et al., 2018; Goodman et al., 2003, 2017; Wetherall et al., 2019; Pössel et al., 2022).

Ostracism may lower subjective social status in several ways. Social exclusion weakens individuals' social networks and support, and limits their access to resources and opportunities (Zhao and Zhang, 2021). Students who are frequently ignored by peers may come to see themselves as belonging to a lower social stratum, regardless of their objective socioeconomic status. Drawing on social comparison theory (Festinger, 1954), ostracized students are likely to make upward comparisons with more accepted peers, which can accentuate perceived inferiority and further reduce their subjective social status.

Although subjective social status has been identified as a powerful predictor of health outcomes that often exceeds objective indicators (Rubin et al., 2016; Wolff et al., 2010), only a few studies have explored its role in the context of psychological crisis vulnerability. Even fewer have examined whether subjective social status functions as a mediator between ostracism and crisis vulnerability in college student populations. Subjective social status may therefore represent a downstream social-cognitive pathway linking interpersonal exclusion to perceived disadvantage, stress exposure, and psychological crisis vulnerability.

### 2.4 Integrating self-uncertainty and subjective social status

Self-uncertainty and subjective social status are likely to be interconnected rather than independent processes. Conceptually, self-uncertainty reflects an immediate internal response to social exclusion, whereas subjective social status represents a more stabilized evaluation of one's social position that may emerge from prolonged uncertainty and repeated social comparison. According to uncertainty–identity theory, when individuals feel uncertain, they may seek identification with groups that mirror their perceived circumstances, including lower-status groups, in order to reduce uncertainty (Hogg, 2007, 2009). High self-uncertainty can therefore bias social comparisons and lead individuals to rely heavily on others' evaluations when judging their social position (Kraus et al., 2012).

Consistent with Bandura (1978) triadic reciprocal determinism, environmental factors (e.g., ostracism), personal factors (e.g., self-uncertainty), and social outcomes (e.g., perceived social status) may influence each other in a dynamic way. Ostracism, as a chronic interpersonal stressor, may first increase self-uncertainty, which then contributes to a lower subjective social status. In turn, low subjective social status may heighten psychological crisis vulnerability by exposing individuals to greater perceived stress, fewer resources, and a sense of disadvantage.

To date, however, there is a lack of formal causal and mediational models that integrate all four constructs—ostracism, self-uncertainty, subjective social status, and psychological crisis vulnerability—within a single framework. The present study addresses this gap by testing a multiple mediation model in which self-uncertainty and subjective social status operate both independently and sequentially in the relationship between ostracism and psychological crisis vulnerability among Chinese college students.

## 3 The present study and hypotheses

Based on the above literature, we propose a model in which ostracism, as an environmental factor, increases psychological crisis vulnerability both directly and indirectly through self-uncertainty and subjective social status. Specifically, we test the following hypotheses:

H1: Ostracism is positively associated with psychological crisis vulnerability among Chinese college students.

H2: Self-uncertainty and subjective social status each independently mediate the relationship between ostracism and psychological crisis vulnerability.

H3: Self-uncertainty and subjective social status jointly form a chain mediation pathway, such that ostracism increases self-uncertainty, which in turn reduces subjective social status, thereby elevating psychological crisis vulnerability.

**Figure 1:**
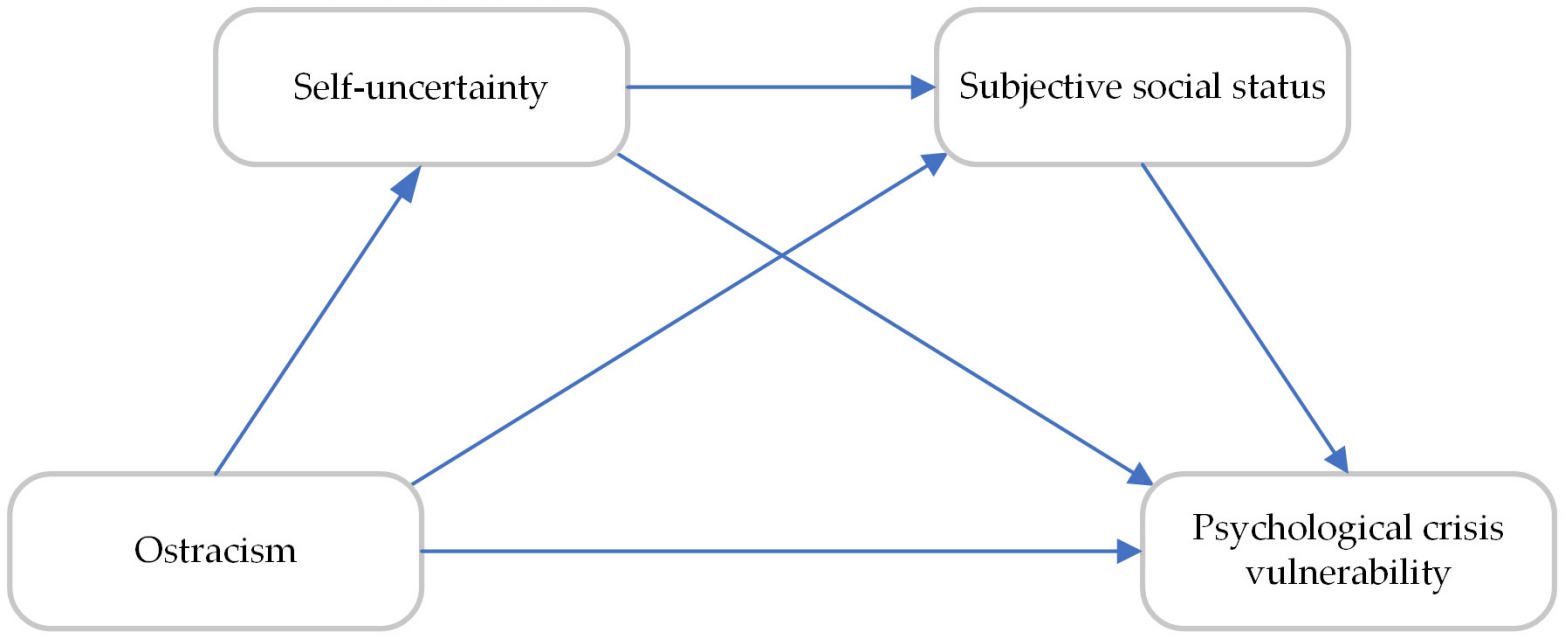
Hypothesized model of ostracism and psychological crisis vulnerability.

The hypothesized multiple mediation model is presented in Figure 1.

## 4 Materials and methods

### 4.1 Participants and Procedure

Participants were recruited through convenience sampling from four comprehensive universities located in economically diverse regions of China, including developed areas in Eastern China and less developed areas in Western China. Participants represented a broad range of majors, including STEM, humanities and social sciences, and arts. A total of 800 college students initially participated in the questionnaire survey. After excluding incomplete or invalid responses, 758 valid questionnaires were retained for the final analyses.

Participants ranged in age from 17 to 26 years (*M* = 19.11, *SD* = 1.48). The sample included 206 males (27.18%) and 552 females (72.82%). Regarding academic year, 582 participants were 1st-year students (76.78%), 70 were 2nd-year students (9.23%), 53 were 3rd-year students (6.99%), and 44 were 4th-year students (5.80%).

The study protocol was reviewed and approved by the Ethics Review Committee of Qujing Normal University. All participants provided written informed consent prior to participation.

### 4.2 Measures

#### 4.2.1 Psychological crisis vulnerability

The Psychological Crisis Vulnerability Scale, developed by Guo et al. (2018), was employed in the present study. This scale comprises 22 items that assess four dimensions: challenge, coping, support, and resilience. Participants rated the items on a 5-point Likert scale, with higher scores indicating greater vulnerability to psychological crisis in response to unexpected events. In this study, the scale demonstrated acceptable internal consistency, with a Cronbach's alpha of 0.84.

#### 4.2.2 Ostracism

The ostracism questionnaire developed by Wu et al. (2013) was utilized in the study. The questionnaire consists of two sub-scales: direct exclusion (10 items) and indirect exclusion (9 items), totaling 19 items. Participants rated the items on a 5-point scale, with higher scores indicating greater perceived exclusion. In this study, the questionnaire demonstrated excellent internal consistency, with a Cronbach's alpha of 0.95.

#### 4.2.3 Self-uncertainty

A 7-item self-uncertainty measure, adapted from Rast III et al. (2013), was utilized in the present study. Participants rated the extent to which they feel concerned, uncertain or worried about themselves and their future. An example item is, ‘I am concerned about my future'. The scale demonstrated acceptable internal consistency, with a Cronbach's alpha of 0.81.

#### 4.2.4 Subjective social status

The MacArthur Subjective Social Status Scale (Adler et al., 2000), a widely used measure of perceived social standing, was employed in this study. The scale uses a 10-rung ladder as a metaphor for social hierarchy, in which participants were asked to assess their perceived position within the social hierarchy. A score of 1 represents the lowest rung (the bottom of the hierarchy), while a score of 10 represents the highest rung (the top of the hierarchy).

#### 4.2.5 Data processing

Data analyses were conducted using SPSS version 23.0. Descriptive statistics were first performed, followed by chain mediation analyses using Model 6 of the PROCESS macro (version 4.1). Gender, age, and academic major were included as covariates in all regression analyses to control for their potential confounding effects.

Common method bias was examined using Harman's single-factor test. An unrotated principal component factor analysis was conducted on all measurement items. Eight factors with eigenvalues greater than one were extracted, and the first factor accounted for 28.76% of the total variance, which was below the recommended threshold of 40%, indicating that common method bias was not a serious concern in this study.

## 5 Results

### 5.1 Descriptive statistics

Table 1 presented the means and standard deviations of the variables, along with the correlation coefficients. The results indicated that ostracism was significantly positively correlated with the psychological crisis vulnerability and the self-uncertainty, while it was negatively correlated with the participants' subjective social states. Additionally, psychological crisis vulnerability was significantly positively correlated with the self-uncertainty and significantly negatively correlated with subjective social states. Furthermore, self-uncertainty was significantly negatively correlated with their subjective social states.

**Table 1:** Means, standard deviations and correlation between the variables.

**Variables**	**Self-uncertainty**			**Subjective social states**			**Psychological crisis vulnerability**		
	**β**	***SE***	***t***	**β**	***SE***	***t***	**β**	***SE***	***t***
Gender	0.07	0.08	1.94	−0.08	0.08	−2.14^*^	0.07	0.07	2.21^*^
Age	0.01	0.04	0.22	−0.05	0.04	−0.80	−0.10	0.04	−1.83
Grade	0.03	0.06	0.45	−0.003	0.06	−0.05	0.07	0.05	1.28
Major	0.11	0.04	3.17^**^	−0.08	0.04	−2.03^*^	−0.02	0.04	−0.56
Ostracism	0.28	0.04	8.05^***^	−0.14	0.04	−3.80^***^	0.39	0.03	12.08^***^
Self-uncertainty				−0.15	0.04	−3.99 ^***^	0.12	0.03	3.76^***^
Subjective social states							−0.25	0.03	−7.83^***^
*R* ^2^	0.09			0.07			0.30		
*F*	15.51			9.02			46.83		

^*^ *p* < 0.05 ^**^ *p* < 0.01^***^ *p* < 0.001. ^*^ *p* < 0.05; ^**^ *p* < 0.01.

### 5.2 The relationship between ostracism and psychological crisis vulnerability: multiple mediating model

All the variables were standardized, with the exception of gender, age, grade, and major. Ostracism was treated as an independent variable, while the psychological crisis vulnerability variable was considered the dependent variable. Self-uncertainty values and subjective social states served as mediating variables. A multiple hierarchical regression analysis was conducted following Model 6 of the Process program (version4.1), facilitating an integrated test of the chain mediation model.

All the variables were standardized, with the exception of gender, age, grade, and major. Ostracism was treated as an independent variable, while the psychological crisis vulnerability variable was considered the dependent variable. Self-uncertainty values and subjective social states served as mediating variables. A multiple hierarchical regression analysis was conducted following Model 6 of the Process program (version4.1), facilitating an integrated test of the chain mediation model.

**Figure 2:**
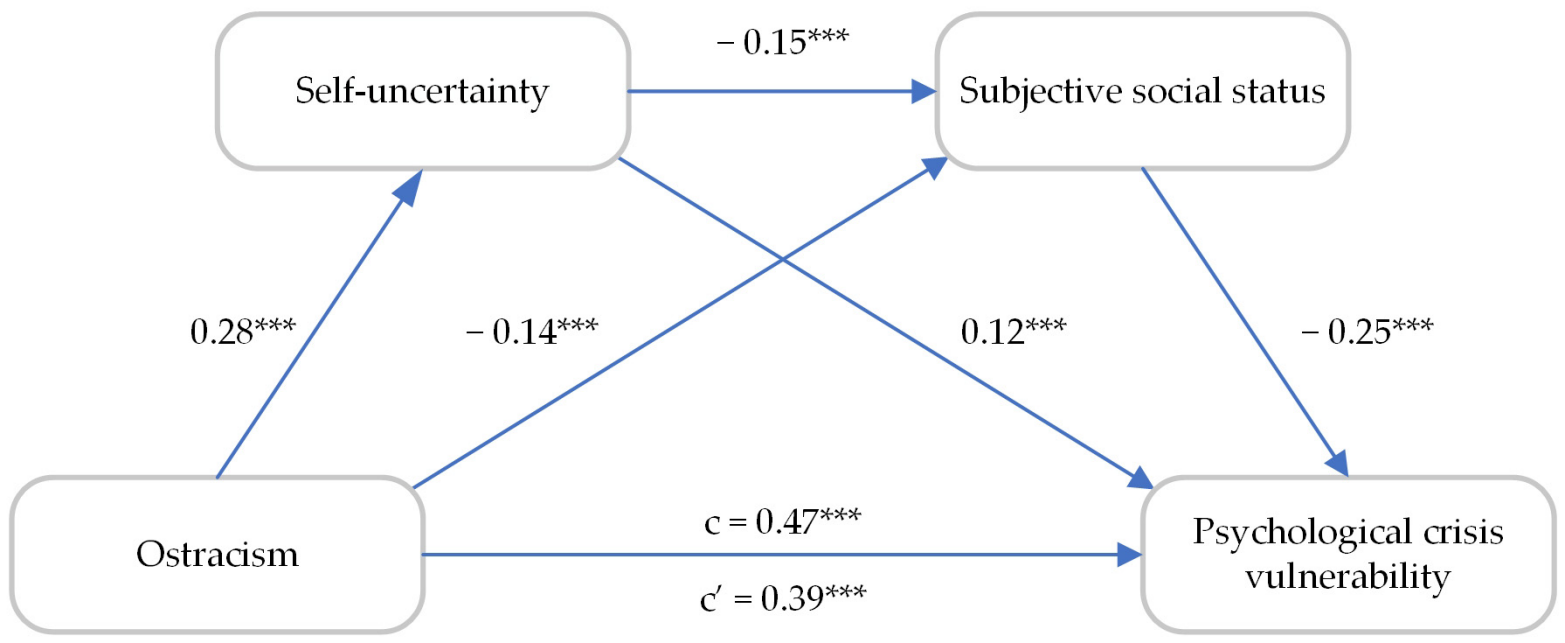
Multiple mediating model of ostracism and psychological crisis vulnerability. *** *p* < 0.001.

As shown in Table 2, under the control of gender, age, grade, and major, the path coefficient from ostracism to self-uncertainty was significant (β = 0.28, *p* < 0.01), and the path coefficient from ostracism to subjective social states was significant (β = −0.14, *p* < 0.01). The path coefficient from self-uncertainty to subjective social states was significant (β = −0.15, *p* < 0.01), and the path coefficient from self-uncertainty to psychological crisis vulnerability was also significant (β = 0.12, *p* < 0.01). The path coefficient from subjective social states to psychological crisis vulnerability was significant (β = −0.25, *p* < 0.01). The effect of ostracism on psychological crisis vulnerability was still significant, after adding self-uncertainty and subjective social states to the test. The model is seen in Figure 2.

**Table 2:** Multiple mediating model between ostracism and psychological crisis vulnerability (*N* = 758).

**Variables**	**1**	**2**	**3**	**4**	**5**	**6**	**7**	**8**
1 Gender	-							
2 Age	−0.069	-						
3 Grade	−0.056	0.808^**^	-					
4 Major	−0.255^**^	0.076^*^	0.074^*^	-				
5 Ostracism	−0.058	0.026	0.027	0.020	-			
6 Psychological crisis vulnerability	0.071	−0.017	0.014	0.001	0.461^**^	-		
7 Self-uncertainty	0.022	0.045	0.049	0.105^**^	0.279^**^	0.277^**^	-	
8 Subjective Social states	−0.051	−0.061	−0.054	−0.077^*^	−0.179^**^	−0.341^**^	−0.199^**^	-
*M*	-	-	-	-	30.468	53.807	38.875	5.639
*SD*	-	-	-	-	12.137	13.942	10.494	1.469

^*^p < 0.05; ^**^p < 0.01.

Furthermore, we employed the Bootstrap method to calculate 95% confidence intervals for each of the 5000 repeated samples, with the results presented in Table 3. The findings indicated that none of the confidence intervals for the tested paths included a value of 0. The direct effect of ostracism on psychological crisis vulnerability among college students was 0.39, while the total indirect effect (i.e., the sum of the three mediated path effect values) was 0.08.

**Table 3:** Direct and mediated effects between ostracism to psychological crisis vulnerability.

**Effect**	**Effect value (SE)**	**|Effect value/direct effect|**	**95% Confidence interval**
Direct effect	0.39(0.03)		[0.33, 0.45]
Path1	0.03(0.01)	0.08	[0.01, 0.06]
Path2	0.04(0.01)	0.10	[0.01, 0.06]
Path3	0.01(0.003)	0.03	[0.004, 0.02]
Total mediated effect	0.08(0.02)	0.21	[0.05, 0.11]
Total effect	0.47(0.03)		[0.40, 0.53]

path1: ostracism—self-uncertainty—psychological crisis vulnerability; path2: ostracism—subjective social states—psychological crisis vulnerability; path3: ostracism—self-uncertainty—subjective social states—psychological crisis vulnerability.

## 6 Discussion

This study investigated the impact of ostracism on psychological crisis vulnerability among Chinese college students, as well as the underlying mechanisms involved. The findings revealed a significant positive association between ostracism and psychological crisis vulnerability, such that higher levels of ostracism were associated with greater vulnerability. These results are consistent with previous research linking ostracism to psychological crisis and severe mental health outcomes (Chen et al., 2020; Baumeister et al., 2005; Poon and Chen, 2014), underscoring ostracism as a critical risk factor for psychological crisis vulnerability. According to the need–threat model, ostracism threatens fundamental psychological needs, including belonging, self-esteem, recognition, and control (Williams, 2009). When these needs are persistently unmet, individuals are more likely to experience psychological stress and distress, thereby increasing their vulnerability to psychological crisis. Recent integrative reviews further highlight ostracism as a pervasive social stressor with wide-ranging psychosocial and behavioral consequences across contexts, reinforcing its central role in mental health vulnerability (Chen et al., 2025).

In addition to these cognitive and status-related mechanisms, ostracism also has a strong emotional component. Experimental and survey studies have consistently shown that being excluded elicits intense negative affect, such as hurt feelings, sadness, anger, and shame, which may further undermine self-regulation and mental equilibrium (Baumeister et al., 2005; Williams, 2009). More recent evidence further suggests that when such negative emotional states become persistent or poorly regulated, their consequences extend beyond psychological distress. For example, mood swings have been linked to physical health problems such as gastrointestinal diseases (Wang et al., 2024), maladaptive behavioral patterns including addictive smartphone use among college students through emotional exhaustion (Feng and Dou, 2024), and socio-emotional difficulties such as alexithymia and somatization (Ayik et al., 2023). In addition, studies focusing on adolescents and young people indicate that emotion-related capacities, including emotional intelligence and emotion regulation abilities, are closely associated with the risk of suicidal ideation and suicidal behavior (Gómez-Tabares et al., 2023). Although emotional responses were not directly measured in the present study, our findings can be understood within a broader theoretical framework integrating the need–threat model, the interpersonal theory of suicide, uncertainty–identity theory, the looking-glass self, and social comparison perspectives. Taken together, these frameworks suggest that ostracism threatens basic psychological needs and heightens self-uncertainty, which may further bias social comparisons toward lower perceived social status and evoke negative affect. These emotional and cognitive disruptions provide an important psychological context for understanding how ostracism translates into broader impairments in self-concept, social evaluation, and mental health outcomes.

Ostracism can lead to a range of negative outcomes, including anxiety, depression, and aggression (Yang et al., 2021), diminished self-esteem (Lei et al., 2024), and problematic behaviors such as suicidal tendencies (Chu et al., 2017). In the present study, ostracism was positively associated with self-uncertainty and negatively associated with subjective social status. These findings suggest that when college students experience ostracism, they not only endure feelings of loneliness, rejection, and lack of acceptance, which may increase suicidal ideation (Chen et al., 2020), but also experience heightened self-uncertainty, which undermines psychological resilience and adaptability. Increased self-uncertainty may hinder the formation of a stable self-concept, resulting in doubts about self-worth and social relationships (Heine et al., 2006). This state may exacerbate feelings of isolation and helplessness, creating a self-reinforcing cycle. Furthermore, ostracism may diminish individuals' perceptions of their social status, thereby adversely affecting their sense of social identity and mental health. Examining the role of self-uncertainty therefore helps clarify the mechanisms through which ostracism affects mental health and may inform potential intervention strategies.

Previous research has shown that subjective social status can capture nuanced aspects of social standing that are not easily reflected by objective indicators, and that it is strongly associated with individual health outcomes (Singh-Manoux et al., 2005; Goodman et al., 2003). For example, subjective social status has been found to predict wellbeing and mental health (Haught et al., 2015). Consistent with this literature, the present study revealed a significant negative association between subjective social status and psychological crisis vulnerability. This finding indicates that students with lower subjective social status are more vulnerable to psychological crisis, and that such negative psychological experiences may further impair their ability to cope with stress and adversity. Accordingly, enhancing college students' perceptions of their subjective social status, and helping them establish more positive social comparisons and self-identities, may represent important intervention strategies for reducing psychological crisis vulnerability.

The findings of this study further indicate that the impact of ostracism on psychological crisis vulnerability operates through both direct and indirect pathways. On one hand, ostracism increases self-uncertainty, which in turn heightens psychological crisis vulnerability. According to the looking-glass self theory, an individual's self-concept is gradually formed and continuously refined through social interactions (Cooley, 1902). Individuals construct self-perceptions by observing how others respond to them, with others' evaluations, attitudes, and behaviors serving as a mirror for self-evaluation and identity formation. When individuals experience ostracism, they are likely to receive negative social feedback or the absence of affirmation (Zhu et al., 2025), which undermines positive self-perceptions and increases self-uncertainty, thereby impairing mental health and social adaptation (Jiang et al., 2020; Williams and Nida, 2022). Elevated self-uncertainty may lead to confusion, unease, and anxiety, as well as reduced self-esteem and confidence (Yang et al., 2017). Under conditions of psychological stress, individuals with high self-uncertainty are more susceptible to negative emotional responses and cognitive biases, and often lack effective coping strategies (Boelen et al., 2014), ultimately increasing their vulnerability to psychological crisis.

On the other hand, ostracism may increase psychological crisis vulnerability by lowering individuals' perceived social status. According to social comparison theory, individuals evaluate their own value and status by comparing themselves with others (Festinger, 1954). Upward comparisons may highlight personal shortcomings, whereas downward comparisons can provide temporary psychological comfort (Gerber et al., 2018). When individuals experience ostracism, they are likely to compare themselves with mainstream groups and recognize that they are being ignored or rejected, which fosters feelings of marginalization. This comparison process contributes to a negative perception of social status and diminished perceived social standing (Midgley et al., 2021; Parsons et al., 2021; Perets et al., 2025). As a result, individuals may perceive themselves as belonging to a lower social tier. Low perceived social status is associated with greater life stress, fewer resources, and reduced social support (Demakakos et al., 2008), making individuals more prone to helplessness and psychological distress. Moreover, low subjective social status is closely intertwined with heightened perceptions of social inequality and unfairness (Hajdu, 2025), which may further undermine individuals' outlook on their future. Collectively, these factors contribute to increased psychological crisis vulnerability.

Furthermore, the results indicate that self-uncertainty and subjective social status form a chain mediating pathway between ostracism and psychological crisis vulnerability. Specifically, ostracism increases self-uncertainty, which is associated with lower subjective social status, ultimately heightening psychological crisis vulnerability. Bandura (1978) triadic reciprocal determinism posits that individual behavior, environmental factors, and personal factors interact dynamically. Within this framework, ostracism can be understood as an external social environmental stressor that shapes individuals' psychological states and behavioral responses (Chen et al., 2025). Ostracism has been shown to undermine individuals' confidence in their own abilities and effectiveness, as reflected in reduced self-efficacy, which may, over time, contribute to heightened self-uncertainty (Rajchert et al., 2024). This self-uncertainty, arising from disrupted self-perceptions following ostracism, leads individuals to question their qualities and social value (Wirth et al., 2024). In turn, individuals are more likely to reassess their social standing, resulting in lower subjective social status (Hogg, 2007, 2009; Kraus et al., 2012). Reduced subjective social status is associated with greater psychological stress and fewer psychosocial resources (Adler et al., 2000; Rahal et al., 2020; Zhao and Zhang, 2021), which exacerbate emotional distress and increase vulnerability to psychological crisis (Wetherall et al., 2019; Pössel et al., 2022).

In summary, this study provides a comprehensive examination of the negative impacts of ostracism on Chinese college students' psychological crisis vulnerability. These impacts extend beyond self-perception to include evaluations of subjective social status and indirect effects through self-uncertainty. Accordingly, institutions of higher education should pay particular attention to students who are vulnerable to ostracism and implement targeted interventions to support their mental health. Interventions aimed at reducing self-uncertainty—such as psychological counseling and structured group activities that foster self-confidence and self-awareness—may be especially beneficial. In addition, helping students develop a more balanced understanding of their subjective social status may promote healthier social comparisons and self-identities. Together, these efforts may contribute to a more supportive campus environment and enhance students' psychological resilience and adaptive coping capacities.

### 6.1 Limitations and future directions

Firstly, the research is limited to a population of Chinese college students, which may restrict the generalizability of the findings to other populations or age groups. Different cultural and social contexts may yield varying results. Secondly, this study employed a cross-sectional design, which didn't offer causal inferences. Longitudinal studies are necessary to establish causal relationships between ostracism, self-uncertainty, subjective social status, and vulnerability to psychological crisis.

Future research should consider expanding the scope of the study to include diverse age groups, social demographics, and cultural contexts to further assess the applicability of the relationship between ostracism and vulnerability to psychological crisis across a broader population. Additionally, conducting longitudinal studies that track the same participants over time would help clarify the causal direction between ostracism and vulnerability to psychological crisis, as well as the dynamic processes of self-uncertainty and subjective social status. Furthermore, intervention studies should be conducted to design targeted interventions for students who are susceptible to ostracism, such as group counseling aimed at reducing self-uncertainty and social practice activities to enhance awareness of subjective social status, and to evaluate the effectiveness of these interventions. In addition, female students were over-represented in our sample. Although we statistically controlled for gender, future studies should strive for more balanced gender ratios or conduct stratified analyses to further verify the robustness of the findings.

Third, because participants were recruited through convenience sampling from only four universities, the sample cannot be considered fully representative of all Chinese college students. Therefore, the generalizability of our conclusions should be interpreted with caution.

Beyond psychosocial pathways, future work should also examine the neurobiological and physiological mechanisms through which ostracism and low subjective social status may increase crisis vulnerability. Prior theoretical and empirical work has shown that social-evaluative threats can trigger heightened cortisol responses and inflammatory processes (Dickerson and Kemeny, 2004), and that social rejection engages neural systems that overlap with physical pain (Eisenberger and Lieberman, 2004). Investigating whether such cortisol and inflammatory responses mediate the long-term impact of ostracism and low subjective social status on self-uncertainty and psychological crisis vulnerability would deepen our understanding of the underlying mechanisms. In addition, future studies could test coping mechanisms and emotional variables (e.g., emotion regulation strategies, negative affect, hopelessness) as additional mediators or moderators, which may help identify targets for interventions designed to buffer the negative impact of ostracism.

## 7 Conclusion

This study systematically explored the mechanisms by which ostracism influences psychological crisis vulnerability among Chinese college students, with a focus on the mediating roles of self-uncertainty and subjective social status. The key findings are as follows:

First, ostracism is a significant positive predictor of psychological crisis vulnerability, consistent with previous research showing that social exclusion threatens basic needs (e.g., belonging, self-esteem) and increases psychological distress. Second, self-uncertainty and subjective social status each partially mediate the relationship between ostracism and crisis vulnerability: Ostracism enhances self-uncertainty (a state of unstable self-concept), which in turn reduces coping ability; meanwhile, ostracism lowers perceived social standing, increasing susceptibility to crisis. Third, a chain mediation effect was confirmed: Ostracism increases self-uncertainty, which further reduces subjective social status, ultimately elevating psychological crisis vulnerability.

These findings advance theoretical understanding by integrating environmental (ostracism) and psychological (self-uncertainty, subjective social status) factors into a unified framework, revealing sequential pathways underlying crisis vulnerability. Practically, they inform targeted interventions in higher education: Reducing ostracism on campus, mitigating self-uncertainty through psychological counseling, and improving students' perceived social status via positive social comparisons may collectively reduce psychological crisis risk. This study thus provides a scientific basis for promoting college students' mental health and preventing crisis events.

## Data Availability

The datasets presented in this study can be found in online repositories. The names of the repository/repositories and accession number(s) can be found below: ScienceDB:10.57760/sciencedb.19563.
